# Identifying young Aboriginal and Torres Strait Islander children in linked administrative data: A comparison of methods

**DOI:** 10.23889/ijpds.v5i1.1100

**Published:** 2020-03-16

**Authors:** BJ McNamara, J Jones, CCJ Shepherd, L Gubhaju, G Joshy, D McAullay, DB Preen, L Jorm, SJ Eades

**Affiliations:** 1 Centre of Epidemiology and Biostatistics, University of Melbourne, Melbourne, Australia; 2 Faculty of Health and Medical Sciences University of Western Australia, Perth, Australia; 3 Telethon Kids Institute, The University of Western Australia, Perth, Australia; 4 Ngangk Yira: Murdoch University Research Centre for Aboriginal Health and Social Equity, Murdoch, Western Australia; 5 National Centre for Epidemiology & Population Health, Australian National University, Canberra, Australia; 6 School of Population and Global Health, University of Western Australia, Perth, Australia; 7 Centre for Big Data Research in Health, The University of New South Wales, Sydney, Australia

## Abstract

**Background:**

In the ongoing debate on optimum methods for identification of Indigenous people within linked administrative data, few studies have examined the impacts of method on population counts and outcomes in family-based linkage studies of Aboriginal children.

**Objective:**

To quantify differences between three algorithms in ascertaining Aboriginal and Torres Strait Islander children in linked administrative data.

**Methods:**

Linked administrative health data for children born in Western Australia (WA) from 2000-2013, were used to examine the cohorts identified by three methods: A) the Indigenous Status Flag (ISF, derived by the WA Data Linkage Branch using a multistage-median approach) for the children alone; B) the ISF of the children, their parents and grandparents; and C) Indigenous status of the child, mother or father on either of the child’s perinatal records (Midwives or birth registration), to determine differing characteristics of each cohort.

**Results:**

Method B established a larger cohort (33,489) than Method C (33,306) and Method A (27,279), with all methods identifying a core group of 26,790 children (80-98%). Compared with children identified by Method A, additional children identified by Methods B or C, were from less-disadvantaged and more urban areas, and had better perinatal outcomes (e.g. lower proportions of small-for-gestational age, 10% vs 16%). Differences in demographics and health outcomes between Methods C and B were minimal.

**Conclusions:**

Demographic and perinatal health characteristics differ by Aboriginal identification method. Using perinatal records or the ISF of parents and grandparents (in addition to the ISF of the child) appear to be more inclusive methods for identifying young Indigenous children in administrative datasets.

**Keywords:**

Aboriginal health, identification, data linkage, Indigenous, child, methodology

## Background

Routinely-collected health and other administrative data are frequently used to report on the health of populations groups [1-3]. Importantly, this type of data is used to identify and track disparities in health between groups [4], to guide policy formulation, program development, service delivery, and allow large-scale evaluation of programs and policies [2, 5]. However, issues around completeness and under-identification of minority populations in these types of data are well-recognised [2, 6], especially for Indigenous peoples, and may have profound effect on reported health outcomes and policy and program decision-making.

Aboriginal and Torres Strait Islander Australians, comprise approximately 3% of the total Australian population [7]. Aboriginal and Torres Strait Islander Australians, are hereafter respectfully referred to as Aboriginal Australians throughout the manuscript. The term ‘Indigenous’ has been used in relation to recorded Indigenous status in the data and the Indigenous status Flag. Morbidity and mortality rates are considerably higher for the Aboriginal Australian population than the total Australian population for many health conditions [8]. Disparities in health outcomes between Aboriginal and non-Aboriginal Australians occur from birth, with higher rates of Aboriginal children born preterm and/or small for gestational age [9, 10], and higher rates of infant and child mortality [11-13]. Tailoring and resourcing of services to improve the health of Aboriginal children and families require accurate information on health outcomes and risk factors at state and regional levels [4, 11, 14]. Given that methods used to identify Aboriginality in data may have differing impacts on the reported outcomes depending on the type of data and on the age and region [4, 15], it is important to examine these potential impacts.

Self-identification is the primary method of identifying Aboriginal and/or Torres Strait Islander persons in Australian data collection systems, although this approach is generally considered to cover only one aspect of legislative and other definitions in use. Commonly, under Australian legistlation, “An Aboriginal or Torres Strait Islander is a person of Aboriginal or Torres Strait Islander descent who identifies as an Aboriginal or Torres Strait Islander and is accepted as such by the community in which he [or she] lives” [16]. Aboriginal people can choose to self-identify differently across diverse settings and over time. They may also self-identify differently from other members of their family. Recording of Aboriginality in routinely-collected health data is predominantly via self-identification. Therefore an individual’s propensity or reluctance to identify in a given situation may depend on previous experiences, on the perceived level of cultural safety, risk of discrimination, bias or differential treatment in different settings, and on broader political and cultural considerations [17, 18]. By consequence, inconsistencies in the recorded Indigenous status for an individual within and between these datasets may legitimately reflect the individual’s choice whether or not to identify as Aboriginal on specific occasions.

Variation in the quality of data collected between datasets and over time also has an impact on reported Aboriginality in the data [2]. A range of factors in the data collection environment can impact on completeness and accuracy, such as: who collects the data, whether and how the question on Aboriginality is asked directly to the person, staff training on this area and the importance given to collection of this data [2]. Under-enumeration of Aboriginal people in administrative datasets is well-recognised as an issue [4, 19, 20]; and the degree of under-identification is reported to vary between states and territories, between types of datasets [15, 19, 20], and over time [20].

For reporting of research and health outcome data, derivation of a single, consistent Indigenous status for each person is often required, to designate a cohort for study and to report longitudinally on individuals. Linkage of administrative datasets allows information from multiple records to be used to improve the identification of Aboriginal people within the data for analysis purposes. However, the choice of algorithm may impact the estimates of health outcomes [4] and impact decisions based on this data. Sensitivity analyses comparing different methods for identifying Aboriginal people in the data are encouraged as best-practice [2]. A 2012 systematic review [15] of such sensitivity analyses, predominantly in adults, found that the choice of method for identifying Aboriginality altered the estimated outcomes for Aboriginal Australians and the disparities with the non-Aboriginal population estimates. These studies compared a range of identification methods, such as the most inclusive (“ever”, identified on a single record), the most restrictive (“always”, identified as Aboriginal every record) and specific algorithms using varying methods based on designated percentages of the records being identified as Aboriginal. The review describes evidence of increasing and decreasing age-standardised disease rates and Aboriginal/non-Aboriginal rate ratios depending on the condition [21, 22], and effects on case-fatality and mortality rates [22, 23], as well as differential effects by age and remoteness of residence [24, 25].

In 2014, the Western Australian Data Linkage Branch (WA DLB) introduced a derived Indigenous status flag (ISF) for individuals, that can be requested for approved research purposes. The flag is created from a number of core datasets held by the WA Department of Health using algorithms developed by the ‘Getting our Story Right’ collaboration [26], and is updated on a regular basis. It uses a multi-stage median approach to create a single consistent status for each individual, designed to maximize the use of available information without undue influence of individual datasets with multiple records per individual [26]. The approach used by the ISF (described in the Methods) has a number of advantages over other, simpler, methods of identification that are sensitive to single misidentification; such as the ‘ever’ and ‘always’ methods that identify a person as Aboriginal if any or all of their records recorded as Indigenous [26] respectively. These methods likely to either over- or under- estimate numbers of Aboriginal people [26, 27]. The development of the flag explored the multi-stage median approach in relation these approaches, other specific percentage-based algorithms and to the original identifier in each of the datasets considered [26]. However, to date there has been little research to assess how the ISF works at different ages and especially for very young children where the numbers of records for each individual are low [26], or how the ISF may be used in data linkage studies where the children under study are clustered within families (i.e. to the same mother) or within multigenerational families.

In this study we compare the numbers and characteristics of children born in WA (2000-2013) who were identified as Aboriginal Australians in WA linked data using three different methods: (A) the derived ISF for the child; (B) the derived ISFs for the child, parents and grandparents; and (C) the recorded Indigenous status in perinatal records for child. These analyses were designed to explore the ISF for use in birth cohorts and other data linkage studies of young children with family connections linkages. The research has informed the cohort selection for one such family-based data linkage study (Defying the Odds).

## Methods

### Participants and study setting

The *Defying the Odds* study is a population-based data linkage study of family and community factors impacting on early child morbidity and mortality in Western Australian Aboriginal children aged 0-<5years [14]. The study cohort is Aboriginal children born in WA in 2000-2013. The state of Western Australia covers the largest land mass of any Australian state or territory (approximately 2.5 million square kilometres). Based on Australian Bureau of Statistics’ Census data, Aboriginal Australians in WA comprise 3.8% of the total WA population, and 13% of the total Australian Aboriginal population [7]. 

### Aboriginal leadership of the research and ethical approvals

Aboriginal researchers (SE, JJ, and DM) led the design of this study as part of the chief investigator’s team for the *Defying the Odds study*, and within the working group for cohort selection. Methods of identification of Aboriginal children within administrative data have been discussed with the *`Defying the Odds’* Aboriginal Community Reference Group and other members of the Aboriginal community. Ethical approval for *Defying the Odds* has been granted by the Western Australian Aboriginal Health Ethics committee (#609) and the WA Department of Health Human Ethics Committee (RGS0000002846, Migrated ID DOH-201530).

### Data sources and Data linkage

*Defying the Odds* Study data comprise 12 linked routinely-collected health and administrative datasets held by the WA Department of Health and the WA Department of Communities – Child Protection and Family Support. These are: WA Midwives' Notification System (MNS); WA Birth Registrations; Hospital Morbidity Data Collection (HMDC); Emergency Department Data Collection (EDDC); Mortality Register; WA Infant, Child and Youth Mortality Database; WA Register of Developmental Anomalies (Birth Defects & Cerebral Palsy) (WARDBP & WARDCP); WA Notifiable Infectious Diseases Database (WANIDD), WA Department of Communities - Child Protection and Family Support data; Mental Health Information System (MHIS); WA Drug and Alcohol Office data; and WA Electoral Roll records. The latter three datasets are available in this study for relatives and siblings of the cohort only. Geographic location data mapped to 2001, 2006, 2011 Australian Bureau of Statistics boundaries (at a minimum level of Collection District or Statistical Area 1, approximately 200 households) are available for all MNS, HMDC, EDDC and Mortality records for the cohort children. Further information on the study datasets has been previously published [14] and is available at http://www.datalinkage-wa.org.au/.

#### Data linkage

Data were linked for this study through the Western Australian Data Linkage System (WADLS) using probabilistic matching based on full name and address, phonetic compression algorithms and other identifiers (including unit medical record numbers) [28]. The links created undergo continuous quality assurance checks, and have a very high degree of accuracy (it estimated that less than 0.3% of chains contained one or more incorrect links) [28, 29]. Data provided to the researchers has overt identifiers removed and is under strict principles of use in terms of approved physical and technological security, use by authorised persons and for authorised purposes only and is not to be merged with other datasets (refer https://www.datalinkage-wa.org.au/resources/policies/).

#### Family connections linkages

This study also uses the family connections linkages contained within WADLS to identify the parents, grandparents and siblings of the cohort children. These linkages are a supplementary set of links determined through the WADLS data collections that identify parent–offspring relationships by linked information from birth registrations and midwives’ notifications [30].

#### Extraction criteria

The data extraction criteria for *Defying the Odds* enabled all identification methods to be applied to the study data without extraction of the entire non-Aboriginal population [14]. Data were extracted for any individual born in WA between 2000 and 2013 who has ever been identified as Aboriginal and/or Torres Strait Islander in any record of the requested datasets OR who had a parent, or grandparent (maternal and paternal), or a full sibling born between 2000-2013, who has ever been identified as Aboriginal and/or Torres Strait Islander in any of the requested datasets. Data for the parents, grandparents and full- and half-siblings of these individuals were then also extracted.

#### Inclusions and exclusions

Children were included in this analysis if they were born 2000-2013 and had family linkage keys ascertaining at minimum their mother within the study data (n=55,939); 93% of these children also had a father linked, 91% had at least one grandparent linked - 80% had their maternal grandparents linked and 62% had their paternal grandparents linked. From the total extracted data of children born 2000-2013, 3753 children flagged as Indigenous by the ISF in their own records were excluded from analysis as they did not have a mother linked within the data. These children were also missing a WA birth registration and a WA MNS record (suggesting that many may have been either born interstate before residing in WA or only had health service contact while visiting WA).

### Identification methods

This analysis examined three methods of identifying the population cohort of Aboriginal and Torres Strait Islander children from within the extracted data: ***Method A***, derived using the Indigenous Status Flag (ISF; indicating Aboriginal and/or Torres Strait Islander origin of the child); ***Method B***, derived using the ISF of the child, the ISF of the parents or the ISF of the grandparents (identification of child, parents or grandparents resulted in selection by this method); and ***Method C***, derived from perinatal records (Midwives Notification System Record or Birth registration) of the child, indicating that the child, or where the child’s status is missing that of mother or father are of Aboriginal and/or Torres Strait Islander origin.

*Creation of the ISF:* The ISF for an individual, used here in *Method A* and *Method B*, was created by the WA DLB using a multi-stage median algorithm applied to variables indicating Aboriginal and/or Torres Strait Islander status from MNS, Birth Registration, HMDC, EDDC, and Death Registration datasets (https://www.datalinkage-wa.org.au/dlb-services/derived-indigenous-status-flag/). This algorithm produces a single consistent status indicator for each individual. The multi-stage median approach, consists of two steps: 1) First, an Indigenous status from each dataset is derived; for individuals with one record in the dataset, the status recorded is used; for those with two records, the dataset status is recorded as Indigenous if at least one record has the Indigenous status as Indigenous. For individuals with three or more records in each dataset, the individual is recorded as Indigenous for the dataset if the person has two or more records where they have been identified as Indigenous. Only records with a non-missing Indigenous status contribute to the derived ISF. 2) Second, the derived Indigenous status from each of these datasets are then combined using the same principles, such that those with three or more datasets where the derived Indigenous status is recorded as Indigenous in at least two datasets then that person is flagged as being Indigenous [26] (refer to Supplementary Figure 1, a visual diagram of the ISF in Method A and B). Complete collection of the child’s Indigenous status on Birth Registrations was introduced in 2007, and on the MNS in 2012; where available, this child’s status was used to derive the child’s MNS or birth registration status for the DLB ISF, without reference to the parental status. Maternal Indigenous status was available on the MNS throughout the period of study, as was maternal and paternal Indigenous status on the birth registrations.

*Method C*, a **perinatal** identification method, used the Indigenous status of the child, mother and father from the birth registration and from the MNS (mother and child only). Children were selected for using this method if they were recorded as Aboriginal and/or Torres Strait Islander on either their birth registration or MNS record (where child status was available), or if their mother or father are recorded as Aboriginal and/or Torres Strait Islander on either of these records if the child’s status was missing for that dataset.

### Study measures

Parental Aboriginality of the children identified by each of the methods was determined using the DLB ISF for each parent (derived from that *parent’s own records* without reference to any of their childrens’ records). Usual residence at birth was coded to broad Indigenous Regions using the Australian Bureau of Statistics (ABS) 2011 Australian Statistical Geography Standard structure [31] from geo-location data from the individual child’s MNS record (SA1 and SA2 in our data extraction). Data from the earliest EDDC presentation or HMDC admission was used to classify location in cases with missing data in MNS. SA1-level social disadvantage and remoteness of residence was determined using the ABS Index of Relative Social Disadvantage [32] and Remoteness Area [33]. Gestational age was determined using antenatal and neonatal clinical indicators of gestational duration [34]. Prematurity was categorized according to World Health Organization standards. Small for Gestational Age (SGA) was determined using the birth weight obtained from the MNS and defined as those children with birth weight less than the 10th percentile of birth weight for their sex and gestational age using national distributions [35]. Low birth weight was classified as birth weight less than 2,500g [35]. 

### Statistical analysis

Cohort numbers were derived using each of the three defined methods, the percentage overlap was calculated using children identified using any of the three methods as denominator. Proportions of children in each cohort by demographic and perinatal health characteristics were determined and 95% confidence intervals calculated using a binomial distribution. Linear regression was used to assess temporal trends in numbers identified by each method, modelling birth year as the independent variable. Assumptions of linearity and normality of residuals were verified using scatter plots and residual plots respectively. Sensitivity analyses removing clear outliers (in Method C) were conducted to assess the impact on estimates of the linear trend. The number of children identified as Aboriginal by birth year as a proportion of total annual births in WA (derived from ABS birth registration data) were calculated for each identification method. Generalised linear models assuming a Poisson distribution were used to model the annual number of births identified as Aboriginal, with total annual births in WA used as an offset. Rate ratios for percentage Aboriginal identification each year relative to that in year 2000 were estimated for each of the identification methods. Analyses were conducted using SAS 9.4 (SAS Institute Inc, Cary, NC, USA) and Stata 16 (StataCorp, College Station, TX).

## Results

### Cohort numbers and Aboriginality of parents

**Figure 1: Numbers of children in the identified cohorts by Method A, B, and C and those uniquely identified by each method.**
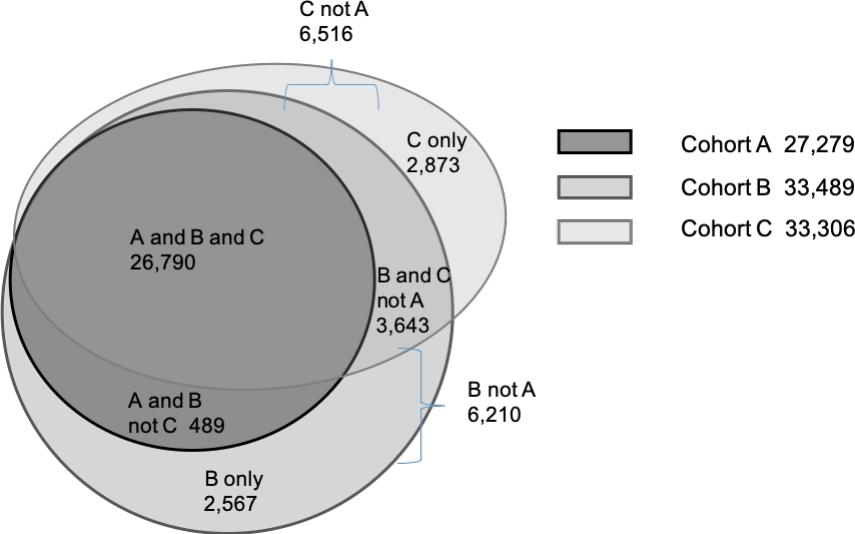
*Method A:* WA Data Linkage Branch (DLB) Indigenous Status Flag for the child; *Method B:* WA DLB ISF for child, ISF for parents or ISF for grandparents; *Method C:* Indigenous status of child or mother or father on Midwives Notification record or WA Birth registration.

Method B established a larger cohort (33,489) than Method C (33,306) or Method A (27,279), with all methods identifying a core group of 26,790 children (80-98%) (Figure 1). A total of 36,362 children were identified by any of the methods. By definition, all children identified by Method A (ISF of child) were identified by Method B (ISF of child, parent, grandparents), and all but 489 children identified by Method A were also identified by Method C (perinatal definition). Of the children identified by Method B, 91% were also identified by Method C, and vice versa, of the children identified by Method C, 91% were identified by Method B. Method B and C identified 6210 and 6516 children, respectively, that were not identified by A; with 3643 of these common to both B and C.

The proportions with family connections linkages available were similar for the each of the cohorts identified as Aboriginal compared to the overall cohort of children included for study - 89-91% in Cohort A, B C had fathers linked and 95-92% in cohort A, B, C had at least one grandparent linked (Supplementary Table 1).

Higher proportions of those identified by the child’s ISF only (Method A) had both Indigenous mothers and fathers than those identified by Methods B and C. Methods B and C identified a higher proportion of children with only the father identified as Indigenous (14.6% and 12.5%) than Method A (6.5%) (Figure 2, Supplementary Table 2). The same was true where the child was identified as Indigenous but the parents were not identified by their own ISF; Method B (7.2%) and Method C (11.1%) compared to Method A (2.2%).

**Figure 2: Aboriginality of mother and father (derived from the Indigenous Status Flag from the parents own records) by three different methods for identifying Aboriginal and/or Torres Strait Islander children:**
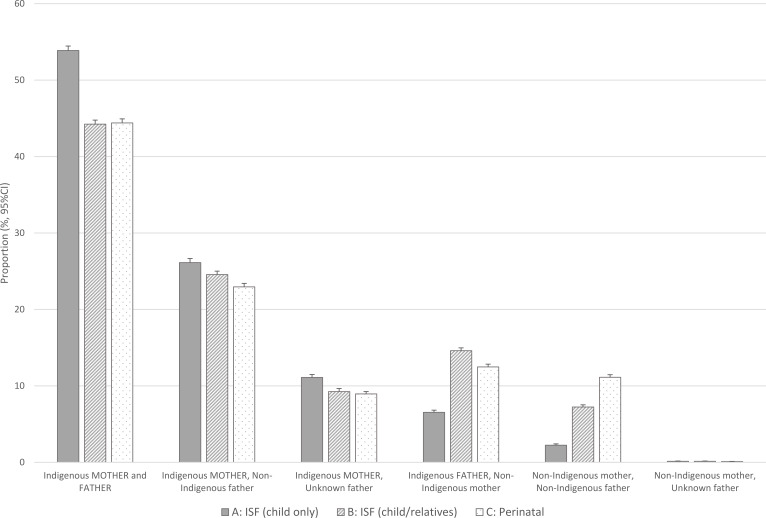
*Method A:* WA Data Linkage Branch (DLB) Indigenous Status Flag for the child; *Method B:* WA DLB ISF for child, ISF for parents or ISF for grandparents; *Method C:* Indigenous status of child or where unavailable, of the mother or father on Midwives Notification record or WA Birth registration.

**Table 1: Proportion, % (number), in each demographic of the children identified as Aboriginal and Torres Strait Islander by identification method A, B and C, and the additional children identified by B or C compared to A.** *Child status where available, majority complete from 2007 on Birth registration and 2012 on

Description	Method A: Child ISF (n=27,279)	Method B: Child or parent or grand-parent ISF (n=33,489)	Method C: MNS or Birth reg. (Child* or parent status) (n=33,306)	B not A (n=6210)	C not A (n=6516)
Year of birth					
2000 - 2001	13.19 (3599)	12.98 (4346)	12.90 (4296)	12.03 (747)	11.80 (769)
2002 - 2003	13.34 (3638)	12.91 (4323)	12.76 (4249)	11.03 (685)	10.51 (685)
2004 - 2005	13.69 (3735)	13.43 (4496)	13.32 (4438)	12.25 (761)	11.80 (769)
2006 - 2007	15.11 (4123)	15.08 (5050)	15.13 (5039)	14.93 (927)	15.27 (995)
2008 - 2009	14.85 (4051)	15.15 (5074)	14.68 (4890)	16.47 (1023)	13.78 (898)
2010 - 2011	14.59 (3981)	15.06 (5044)	14.56 (4849)	17.12 (1063)	14.69 (958)
2012 - 2013	15.22 (4152)	15.40 (5156)	16.65 (5545)	16.17 (1004)	22.15 (1443)
ISRD (Aus.) quintiles					
1 -Most disadvantage	49.83 (13592)	45.34 (15185)	45.11 (15026)	25.65 (1593)	24.80 (1616)
2	22.43 (6118)	23.10 (7735)	23.16 (7713)	26.04 (1617)	26.30 (1714)
3	12.76 (3482)	14.07 (4713)	14.01 (4665)	19.82 (1231)	19.54 (1273)
4	6.35 (1731)	7.93 (2655)	8.18 (2723)	14.88 (924)	16.01 (1043)
5 - Least disadvantage	3.05 (832)	4.48 (1499)	4.55 (1515)	10.74 (667)	10.94 (713)
Missing	5.59 (1524)	5.08 (1702)	5.00 (1664)	2.96 (178)	2.41 (157)
Remoteness of Residence					
Major Cities	35.68 (9732)	41.05 (13748)	42.16 (14041)	64.67 (4016)	70.12 (4569)
Inner Regional	5.36 (1462)	6.16 (2062)	5.90 (1966)	9.66 (600)	8.47 (552)
Outer Regional	16.11 (4395)	15.37 (5146)	14.92 (4970)	12.09 (751)	10.07 (656)
Remote	18.02 (4916)	16.17 (5414)	15.90 (5296)	8.02 (498)	6.72 (438)
Very Remote	20.15 (5496)	16.93 (5669)	16.88 (5622)	2.79 (173)	2.32 (151)
Missing	4.68 (1278)	4.33 (1450)	4.24 (1411)	2.77 (172)	2.30 (150)
Indigenous Region					
Broome	6.88 (1876)	6.04 (2022)	5.98 (1993)	2.35 (146)	2.16 (141)
Geraldton	10.64 (2903)	9.53 (3192)	9.27 (3089)	4.65 (289)	3.55 (231)
Kalgoorlie	7.69 (2099)	7.12 (2385)	7.09 (2362)	4.61 (286)	4.42 (288)
Kununurra	8.57 (2337)	7.13 (2387)	7.12 (2372)	0.81 (50)	0.72 (47)
Perth	34.53 (9419)	39.49 (13225)	40.79 (13585)	61.29 (3806)	67.34 (4388)
South Hedland	9.62 (2625)	8.67 (2905)	8.49 (2828)	4.51 (280)	3.45 (225)
South-Western	13.69 (3735)	14.96 (5009)	14.23 (4738)	20.52 (1274)	17.28 (1126)
West-Kimberley	6.84 (1867)	5.69 (1907)	5.67 (1887)	0.64 (40)	0.46 (30)
Missing	1.53 (418)	1.36 (457)	1.36 (452)	0.63 (39)	0.61 (40)

### Demographics of the cohorts

The numbers of children identified as Aboriginal increased over time in all cohorts, (p<0.006, with the exception of those in C not in B). A linear increase of 24 (95%CI 14, 34) and 40 (26, 53) Aboriginal births per year were observed in Cohort A and B respectively.

The numbers of children in Cohort C increased by approximately 48 (31, 65) births per year. However, the assumption of linearity was not strictly observed in this cohort due to a jump in proportion of children born in 2012-2013 (Table 1) . Analysis considering 2012-2013 births as an outlier showed and linear increase of 38 (95%CI 17, 58) births per year. The increased numbers in Cohort C born in 2012 -2013 coincides with the introduction of the child’s Indigenous status on the MNS (64% of children in C not B (528/824) born in these years were identified based on this identifier), as such the increased numbers identified as Aboriginal are likely to reflect a step-change in identification by this method that will persist rather than being isolated outliers.

While the numbers of children identified as Aboriginal by each method increased over the study period, the increase was not reflected as an increase in the proportion of the total births in WA. Compared to the proportion of births identified as Aboriginal in 2000 (7.2%, 8.7%, and 8.5% for Method A, B, and C respectively), lower proportions were identified as Aboriginal by each of the identification methods for the years 2008 onwards, with the exception of years 2012 and 2013 for Method C (data available on request).

Overall, children identified by Method A were more likely to reside in areas of greater socio-economic disadvantage and remote and very remote areas (Table 1); with higher proportions of the Method A cohort who resided in Broome, Geraldton, Kununurra, and West Kimberley. Clear differences are evident when examining residence of the additional children identified by B and C (but not by A) - 61% and 67% of these children resided in Perth compared to 35% in the Method A cohort, and only 25-26% of these children not identified by Method A resided in the most disadvantaged areas compared to 50% in the Method A cohort.

### Perinatal health of the cohorts

Among Method A children, there were higher proportions of liveborn infants who had low birth weight (14%) and who were small for gestational age (16%) than in the cohorts derived from Methods B and C (12% and 15% respectively, Table 2). Further, the additional children identified as Aboriginal by Methods B and C that were not identified by A, had better perinatal outcomes than those identified by Method A – with greater proportions of liveborn infants born with Apgar scores of 9 or above at 1 minute (60% or 61% vs. 57%), and proportionally fewer being born at low birth weight (7% vs. 14%), SGA (10% vs. 16%) and preterm (with lower proportions of extremely, very, moderately-late preterm births in these additionally identified children).

**Table 2: Perinatal outcomes – Proportion, % (number), of the children identified as Aboriginal and Torres Strait Islander by identification method A, B and C, and the additional children identified by B or C compared to A.** *Child status where available, majority complete from 2007 on Birth registration and 2012 on MN. SD (standard deviation).

	Method A: Child ISF (n=27,279)	Method B: Child or parent or grand-parent ISF (n=33,489)	Method C: MNS or Birth reg. (Child* or parent status) (n=33,306)	B not A (n=6210)	C not A (n=6516)
Data available					
Midwives Notification record	99.83 (27233)	99.85 (33439)	99.85 (33256)	99.94 (6206)	99.94 (6512)
Birth registration record	88.76 (24213)	90.63 (30352)	90.95 (30293)	98.86 (6139)	99.29 (6470)
Gestational Age, (weeks)					
mean (SD)	38.1 (2.9)	38.2 (2.8)	38.2 (2.8)	38.6 (2.3)	38.6 (2.2)
Missing	0.5 (130)	0.4 (139)	0.4 (141)	0.1 (9)	0.2 (11)
Infant Birthweight (g)					
mean (SD)	3116.6 (713.2)	3153.1 (699.9)	3156.1 (698.2)	3313.6 (613.4)	3321.8 (602.7)
Missing	0.2 (63)	0.2 (71)	0.2 (68)	0.1 (8)	0.1 (5)
Preterm birth					
Extremely Preterm (<28 weeks)	1.81 (493)	1.64 (548)	1.59 (530)	0.89 (55)	0.68 (44)
Very Preterm (28-32 weeks)	1.72 (469)	1.56 (522)	1.59 (529)	0.85 (53)	1.01 (66)
Moderate -Late Preterm 32-37 weeks	11.89 (3244)	11.05 (3699)	10.94 (3644)	7.33 (455)	7.17 (467)
Term (>37 weeks)	84.10 (22943)	85.34 (28581)	85.46 (28462)	90.79 (5638)	90.98 (5928)
Missing	0.48 (130)	0.42 (139)	0.42 (141)	0.14 (9)	0.17 (11)
Live born infant characteristics					
Apgar score of >=9 at 1 min	57.33 (15394)	57.77 (19071)	58.11 (19090)	59.69 (3677)	61.16 (3963)
Low birth weight (<2500g)	13.53 (3634)	12.31 (4065)	12.24 (4022)	7.00 (431)	6.79 (440)
SGA (<10th percentile)	16.44 (4414)	15.31 (5053)	15.15 (4976)	10.37 (639)	9.71 (629)

*Differences between children identified in cohorts B and C:* Both Method B and C identified children that were not identified by A. There were also children that were uniquely identified by B or by C (n=3056 were by B not C; and n=2873 were by C not B; Supplementary Table 3 and 4). Compared to the full cohorts of Method B or C, higher proportions of each of these additionally identified children resided in the least socio-economically disadvantaged areas (11.6 – 12.9% in these additionally identified children vs 4.5-4.6% in the full cohorts of B and C). Similarly, both of these groups of additionally identified children had higher proportions who resided in major cities, with a lower proportion in remote and very remote areas. This was especially the case for the additional children identified by C not B (5.3% remote and 2.3% very remote) compared with those identified by B not C (8.8% remote and 3.6% very remote), and certainly compared to the full B and C cohorts (15.9-16.2 remote and 16.88-16.93% very remote). Those children identified uniquely by Method C (not Method B) had higher proportions with Apgar >=9 at 1 min than those identified by Method B and not C (Supplementary Table 4). Both these groups had better perinatal outcomes than the Method A cohort and the full cohorts of Method B and C.

## Discussion

This study found differences in the numbers and characteristics of Aboriginal children identified by the three Methods examined. The study demonstrates how the new WA DLB ISF derived by a multi-stage median approach compares to identification of children via the child’s perinatal records, as well as the effect of additional identification of children using the ISF of parents and grandparents, on the numbers and characteristics of those identified. We found that the cohort of young Aboriginal children identified by DLB ISF from their own records alone was the most restricted in number and resided in areas of greater socio-economic disadvantage, and more remote areas than those identified by the methods also including the ISF of their relatives or the method using identification on the child’s perinatal records. Perinatal health outcomes were worse for those identified by the ISF of the child’s own records than the other two methods examined. The methods using the child’s perinatal records or using the ISF of children, their parents and grandparents appear to increase the identification of children from higher socio-economic areas and of children with only one parent who is Aboriginal and/or Torres Strait Islander, especially the father.

Aboriginal children from families residing in areas of least disadvantage and where the father alone is Aboriginal are groups reported to be most likely to be under identified in administrative data. A comparison of identification of Aboriginal children within routinely-collected data (MNS and birth registrations) to identification in population-based survey data from the WA Aboriginal Child Health Survey (conducted in 2000-2002), found that children identified by the survey but not the administrative datasets were “more likely to be living in urban areas, in less disadvantaged areas, and to have only one parent who identifies as being of Aboriginal and/or Torres Strait Islander origin, particularly the father… and also more likely to have better health and wellbeing outcomes” [5]. The non-random distribution of health and demographic characteristics that we have observed in the children identified by the different methods parallel those of this prior study. It is not possible to determine if the additional families identified in our study by Method B and Method C compared to Method A are correctly identified. However, the considerable number of children additionally identified who were common to both Methods B and C (n=3643) suggests that identification by these two methods, may be more inclusive of these specific-groups of Aboriginal children.

Systematic differences in the characteristics of the children identified as Aboriginal within routinely-collected data according to the method of ascertainment have important policy implications, because it potentially affects reported health outcomes and measures of inequality used to inform resource allocation decisions and monitoring of the impacts of public health and health care investments. This study has shown differences in the rates of SGA, low birth weight and prematurity for the cohorts depending on the identification method chosen. Similarly, modeling by Lawrence et al. to adjust population estimates from 1980-2006 for under-identification, demonstrated improvements in the population estimates of neonatal outcomes for Aboriginal births, but showed that the patterns in the time series remained unchanged over that time period [5]. Inclusion of more Aboriginal children from higher socio-economic backgrounds in urban areas may also have implications for reports of within-population variation in health outcomes for Aboriginal children across regions, potentially increasing reported urban-remote differences in health outcomes [5, 36].

This study highlights the impact that changes in the collected Aboriginality data can have on the numbers of children identified as Aboriginal. Method C identified a higher proportion of children in 2012 and 2013 than the other methods, as a result of introduction the Indigenous status of the child on the MNS, introduced routinely in WA in 2012. An additional 528 children born in 2012-2013, not identified on their DLB ISF or through their parents and grandparents ISF, were identified by Method C via the Indigenous status for the child on the MNS. The vast majority of these children (n=523) had a birth registration available on which they themselves were not identified as Indigenous. This increased identification following the introduction of this Indigenous status indicator, suggests that there has been a step change in the identification by Method C, and that the demographics of the additional children identified by these algorithms may differ pre- and post 2012. Caution should be taken, and differential identification should be considered as a partial explanation when reporting of changes in health outcomes in WA for Aboriginal children for time periods including both pre- and post- 2012, especially when perinatal identification methods have been used.

Our study was restricted to children who have a linkage to their mother within the data. Maternal-child linkages in WADLS are created from data on both the MNS and/or birth registration. The MNS is required to be collected by law by midwives for all births in WA greater than 20 weeks gestation (live and stillbirth), therefore maternal information from the MNS is available for nearly all children born in WA. Unregistered birth or delayed registration may impact on the availability of birth registration and paternal linkages [37]. In the total population, missing paternal information on birth registrations has been shown to be associated with greater social disadvantage, younger maternal age and poor birth outcomes [37, 38]. In our study, the additional children identified by Methods B and C (B not A, and C not A) had higher proportions with a birth registration and paternal linkage (99.9 and 99.3%) compared to Cohort A (88.8%). By contrast, the proportions with linkage to a grandparent were slightly lower in these groups than Cohort A (89% and 80%, compared to 94%).

One limitation of the current study is that the comparison of identification methods undertaken relied solely on linked routinely-collected data. Previous studies that have compared Aboriginal identification in cohorts using data from population-based surveys linked to administrative data have demonstrated there is a substantial proportion of under-identification in administrative data [5]. However, the under-ascertainment compared to survey data may be reduced in future validation studies, following the introduction of the child’s Indigenous status to the WA birth registrations and the MNS.

These results focus on identifiers of Indigenous status at birth and within the early years of life. The findings may not be applicable in relation to identification of older Aboriginal people, where birth registration and midwives’ records may be unavailable. In these situations, the ISF for an individual (based on a multi-stage median) is likely to be one of the optimal methods of incorporating information from multiple morbidity-based records from multiple linked sources, in a way that is robust to single misclassification [27]. Also of note, Method B in this study relies on the DLB ISF for the parents, grandparents and child and that identification as Indigenous through any of these ISFs results in the child’s identification in the cohort – there has been no further consideration of the numbers of records or datasets providing information for each person’s ISF. Some misclassification is possible for Method B through family relationships. Adoption is not able to be clearly ascertained from the data – non-Aboriginal children adopted into Aboriginal families may be misclassified, and Aboriginal children adopted into non-Aboriginal families would only be identified if the child themselves was identified as Aboriginal on their own records if adopted parents were recorded on the birth registration.

### Implications for future studies

All three identification methods examined in this paper offer ways of incorporating information from multiple linked records in the presence of family connection linkages. Our findings suggest that Methods B and C are likely to define the most inclusive and reliable study cohort in studies on young children. Differences in identification methods affect reported health outcomes and health disparities; with the inclusion of more Aboriginal children identified in urban and less socio-economically disadvantaged areas some degree of reduction in the health disparities reported between young Aboriginal and non-Aboriginal children may be expected. The choice between these methods will require consideration on an individual study basis. Method C (perinatal) has advantages in terms of the simplicity of the method, the ready availability of the required data in most Australian states, and the intuitive advantage of not including morbidity-based records in the cohort selection. Arguably, morbidity-based datasets may introduce a bias that offers greater chance for children with higher numbers of morbidity-based datasets to be identified as Aboriginal, however for the ISF algorithm this is only likely to have an influence if child is not identified on any perinatal dataset. However, while Method C is not dependent on morbidity-based records, differential or delayed registration of birth and the availability of paternal information on the registration remains a source of potential bias for this method, as does the risk of misclassification based on a single perinatal identifier. Caution should also be taken in relation to the sensitivity of identification by this method to changes in the availability of Indigenous status indicators on the perinatal records. Method B (ISF child/relatives) utilises all available information, including morbidity- and mortality-related records, but largely avoids the issue for cohort selection in family-based studies of inconsistent identification between siblings; all children to the parent or grandparent are identified as being Aboriginal if their parent or grandparent is identified by their own ISF. With the use of relatives’ records, this method is likely to be beneficial in the identification of specific population groups with few records, such as stillbirths. Method B, like Method A allows flexibility to identify people where there is no birth registration or MNS record or family linkages via their own health service records. In this way, children of Aboriginal fathers who do not yet have their birth registered or Aboriginal children where their father’s status is not recorded on the birth registration may be identified by child’s morbidity-related records included in the ISF.

## Conclusions

In conclusion, research and evaluation using linked health and social data often require a single, consistent Indigenous status to be derived from multiple records for each individual. This study provides information on the numbers and characteristics of Aboriginal children identified within linked routinely-collected administrative data by three different validated methods. These data may promote understanding of the potential impacts of a chosen method on reported health outcomes for Aboriginal Australians across regions. They highlight the importance of considering the effect of methods for identifying Aboriginality, on cohort characteristics and outcomes, and in research, monitoring and evaluation using this type of data.

## Author contributions

Aboriginal researcher SE, and non-Aboriginal researchers BM and LG conceived the study, with scientific input on the design and analysis from Aboriginal researchers JJ and DM and non-Aboriginal researchers CS, GJ, DP and LJ. BM conducted the analysis and wrote the initial draft of the manuscript. All authors contributed to critical revisions of subsequent manuscript drafts and approve of the final version.

## Ethics Statement

Ethical approval for *Defying the Odds* has been granted by the Western Australian Aboriginal Health Ethics committee (#609) and the WA Department of Health Human Ethics Committee (RGS0000002846, Migrated ID DOH-201530), and The University of Melbourne Human Ethics Sub Committee (#1851158).

## Supplementary Appendices

Supplementary Tables & Figures

## Abbreviations

ABS	Australian Bureau of Statistics
AIHW	Australian Institute of Health and Welfare
ARIA+	Accessibility and Remoteness Index of Australia Plus
DLB	Data Linkage Branch
EDDC	Emergency Department Data Collection
HMDC	Hospital Morbidity Data Collection
ISF	Indigenous status flag
MHIS	Mental Health Information System
MNS	Midwives' Notification System
SGA	Small for Gestational Age
WA	Western Australia
WADLS	Western Australian Data Linkage System
WANIDD	WA Notifiable Infectious Diseases Database
WARDBP	WA Register of Developmental Anomalies (Birth Defects)
WARDCP	WA Register of Developmental Anomalies (Cerebral Palsy)
