# Supplementary material for: Identifying young Aboriginal and Torres Strait Islander children in linked administrative data: A comparison of methods
Source: Int J Popul Data Sci. 2020 Mar 16;5(1):1100. doi: 10.23889/ijpds.v5i1.1100 (PMC7473276; doi:10.23889/ijpds.v5i1.1100)
Supplement: Supplementary Tables & Figures [file ijpds-05-1100-s001.pdf]

Supplementary Figure 1: Algorithms for the creation of the WA DLB ISF in Method A and Method B for the derivation of a single consistent Indigenous Status to identify Aboriginal and Torres Strait Islander children.

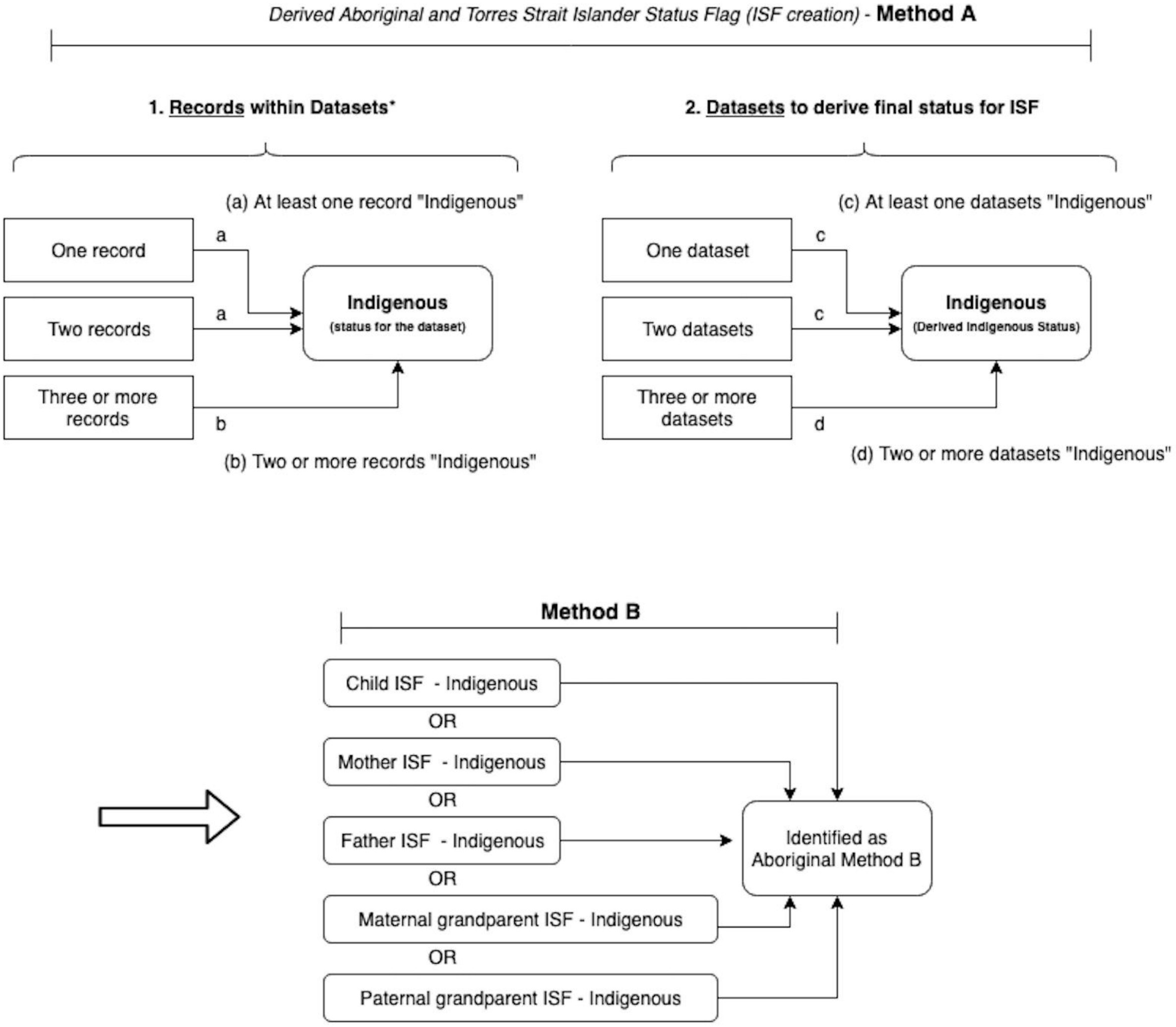

*Datasets used in the derivation of the WA DLB ISF: MNS, Birth Registration, HMDC, EDDC, and Death Registration

Supplementary Table 1: Parent and grandparent linkages available within the data by cohort

		A	B	C	B not A	C not A	B not C	C not B
**Mother**	n	27279	33489	33306	6210	6516	3056	2873
	%	100	100	100	100	100	100	100
**Father**	n	24213	30352	30293	6139	6470	2921	2862
	%	88.76	90.63	90.95	98.86	99.29	95.58	99.62
**Any grandparent**	n	25890	31425	30655	5535	5198	2756	1986
	%	94.91	93.84	92.04	89.13	79.77	90.18	69.13
**All grandparents**	n	14594	17865	17243	3271	2805	1552	930
	%	53.50	53.35	51.77	52.67	43.05	50.79	32.37
**Number of grandparents**								
0	n	1389	2064	2651	675	1318	300	887
	%	5.09	6.16	7.96	10.87	20.23	9.82	30.87
1	n	41	41	41	0	0	0	0
	%	0.15	0.12	0.12				
2	n	11219	13483	13335	2264	2393	1204	1056
	%	41.13	40.26	40.04	36.46	36.72	39.40	36.76
3	n	36	36	36	0	0	0	0
	%	0.13	0.11	0.11				
4	n	14594	17865	17243	3271	2805	1552	930
	%	53.5	53.35	51.77	52.67	43.05	50.79	32.37
**Total**		**27279**	**33489**	**33306**	**6210**	**6516**	**3056**	**2873**

Supplementary Table 2: Aboriginality of mother and father (derived from the Indigenous Status Flag from the parents own records) by three different methods for identifying Aboriginal and/or Torres Strait Islander children (data shown in Figure 2)

	**A: Child ISF (n=27,482)**	**B: Child or parent or grandparent ISF (n=33,697)**	**C: Perinatal Child MNS or Birth reg (Parent or child* status) (n=33,510)**
**Parental Identification (by ISF)**	**% (95%CI) n**	**% (95%CI) n**	**% (95%CI) n**
Indigenous mother and father	53.87% (53.28, 54.46) 14696	44.24% (43.71, 44.77) 14816	44.40% (43.87, 44.93) 14788
Indigenous mother, Non-Indigenous father	26.13% (25.60, 26.65) 7127	24.55% (24.09, 25.01) 8221	22.96% (22.51, 23.41) 7648
Indigenous mother, Unknown father	11.11% (10.74, 11.48) 3031	9.24% (8.93, 9.55) 3094	8.95% (8.65, 9.26) 2982
Non-Indigenous mother, Indigenous father	6.53% (6.24, 6.83) 1782	14.60% (14.23, 14.98) 4891	12.48% (12.12, 12.83) 4155
Non-Indigenous mother, Non-Indigenous father	2.23% (2.05, 2.40) 608	7.24% (6.96, 7.52) 2424	11.12% (10.78, 11.45) 3702
Non-Indigenous mother, Unknown father	0.13% (0.09, 0.17) 35	0.13% (0.09, 0.17) 43	0.09% (0.06, 0.13) 31

*Child status where available, majority complete from 2007 on Birth registration and 2012 on MNS

Supplementary Table 3: Demographics (number, n, and proportion, %) of the children identified as Aboriginal and/or Torres Strait Islander by each identification method (A, B, C – shaded cells) and the additional children identified compared to Methods A, B and C.

**Description**	**Method A: Child ISF (n=27,279) n (%, [95%CI])**	**Child/parent/grand-parent ISF** (not by child ISF) **B not A (n=6210) n (%, [95%CI])**	**Perinatal Definition** (not by child ISF) **C not A (n=6516) n (%, [95%CI])**	**Method B: Child or parent or grandparent ISF (n=33,489) n (%, [95%CI])**	**Method C: Perinatal Child MNS or Birth reg (Parent or child* status) (n=33,306) n (%, [95%CI])**	**Child/parent/grand-parent ISF** (not in Perinatal Definition) **B not C (n=3056) n (%, [95%CI])**	**Perinatal Definition** (not in child/parent/ grandparent ISF**) C not B (n=2873) n (%, [95%CI])**
**Year of birth**							
2000 - 2001	3599 (13.19% [12.79, 13.59])	747 (12.03% [11.22, 12.84])	769 (11.80% [11.02, 12.59])	4346 (12.98% [12.62, 13.34])	4296 (12.90% [12.54, 13.26])	373 (12.21% [11.04, 13.37])	323 (11.24% [10.09, 12.40])
2002 - 2003	3638 (13.34% [12.93, 13.74])	685 (11.03% [10.25, 11.81])	685 (10.51% [9.77, 11.26])	4323 (12.91% [12.55, 13.27])	4249 (12.76% [12.40, 13.12])	375 (12.27% [11.11, 13.43])	301 (10.48% [9.36, 11.60])
2004 - 2005	3735 (13.69% [13.28, 14.10])	761 (12.25% [11.44, 13.07])	769 (11.80% [11.02, 12.59])	4496 (13.43% [13.06, 13.79])	4438 (13.32% [12.96,13.69])	394 (12.89% [11.70, 14.08])	336 (11.70% [10.52, 12.87])
2006 - 2007	4123 (15.11% [14.69, 15.54])	927 (14.93% [14.04, 15.81])	995 (15.27% [14.40, 16.14])	5050 (15.08% [14.70, 15.46])	5039 (15.13% [14.74, 15.51])	453 (14.82% [13.56, 16.08])	442 (15.38% [14.07, 16.70])
2008 - 2009	4051 (14.85% [14.43, 15.27])	1023 (16.47% [15.55, 17.40])	898 (13.78% [12.94, 14.62])	5074 (15.15% [14.77, 15.54])	4890 (14.68% [14.30, 15.06])	522 (17.08% [15.75, 18.42])	338 (11.76% [10.59, 12.94])
2010 - 2011	3981 (14.59% [14.17, 15.01])	1063 (17.12% [16.18, 18.05])	957 (14.69% [13.83, 15.55])	5044 (15.06% [14.68, 15.44])	4849 (14.56% [14.18, 14.94])	504 (16.49% [15.18, 17.81])	309 (10.76% [9.62, 11.89])
2012 - 2013	4152 (15.22% [14.79, 15.65])	1004 (16.17% [15.25, 17.08])	1443 (22.15% [21.14, 23.15])	5156 (15.40% [15.01, 15.78])	5545 (16.65% [16.25, 17.05])	435 (14.23% [13.00, 15.47])	824 (28.68% [27.03, 30.33])
**IRSD (National) - quintiles**							
1 (Most disadvantaged)	13592 (49.83% [49.23, 50.42])	1593 (25.65% [24.57, 26.74])	1616 (24.80% [23.75, 25.85])	15185 (45.34% [44.81, 45.88])	15026 (45.11% [44.58, 45.65])	766 (25.07% [23.53, 26.60])	607 (21.13% [19.64, 22.62])
2	6118 (22.43% [21.93, 22.92])	1617 (26.04% [24.95, 27.13])	1714 (26.30% [25.24, 27.37])	7735 (23.10% [22.65, 23.55])	7713 (23.16% [22.70, 23.61])	719 (23.53% [22.02, 25.03])	697 (24.26% [22.69, 25.83])
3	3482 (12.76% [12.37, 13.16])	1231 (19.82% [18.83, 20.81])	1273 (19.54% [18.57, 20.50])	4713 (14.07% [13.70, 14.45])	4665 (14.01% [13.63, 14.38])	633 (20.71% [19.28, 22.15])	585 (20.36% [18.89, 21.83])
4	1731 (6.35% [6.06, 6.63])	924 (14.88% [13.99, 15.76])	1043 (16.01% [15.12, 16.90])	2655 (7.93% [7.64, 8.22])	2723 (8.18% [7.88, 8.47])	475 (15.54% [14.26, 16.83])	543 (18.90% [17.47, 20.33])
5 (Least disadvantaged)	832 (3.05% [2.85, 3.25])	667 (10.74% [9.97, 11.51])	713 (10.94% [10.18, 11.70])	1499 (4.48% [4.25, 4.70])	1515 (4.55% [4.32, 4.77])	355 (11.62% [10.48, 12.75])	371 (12.91% [11.69, 14.14])
Missing	1524 (5.59% [5.31, 5.86])	178 (2.87% [2.45, 3.28])	157 (2.41% [2.04, 2.78])	1702 (5.08% [4.85, 5.32])	1664 (5.00% [4.76, 5.23])	108 (3.53% [2.88, 4.19])	70 (2.44% [1.87, 3.00])

**Remoteness of Residence**							
Major Cities of Australia	9732 (35.68% [35.11, 36.24])	4016 (64.67% [63.48, 65.86])	4569 (70.12% [69.01, 71.23])	13748 (41.05% [40.53, 41.58])	14041 (42.16% [41.63, 42.69])	1810 (59.23% [57.49, 60.97])	2103 (73.20% [71.58, 74.82])
Inner Regional Australia	1462 (5.36% [5.09, 5.63])	600 (9.66% [8.93, 10.40])	552 (8.47% [7.80, 9.15])	2062 (6.16% [5.90, 6.41])	1966 (5.90% [5.65, 6.16])	327 (10.70% [9.60,11.80])	231 (8.04% [7.05, 9.03])
Outer Regional Australia	4395 (16.11% [15.68, 16.55])	751 (12.09% [11.28, 12.90])	656 (10.07% [9.34, 10.80])	5146 (15.37% [14.98, 15.75])	4970 (14.92% [14.54, 15.30])	434 (14.20% [12.96,15.44])	258 (8.98% [7.93, 10.03])
Remote Australia	4916 (18.02% [17.57, 18.48])	498 (8.02% [7.34, 8.69])	438 (6.72% [6.11, 7.33])	5414 (16.17% [15.77, 16.56])	5296 (15.90% [15.51, 16.29])	269 (8.80% [7.80, 9.81])	151 (5.26% [4.44, 6.07])
Very Remote Australia	5496 (20.15% [19.67, 20.62])	173 (2.79% [2.38, 3.20])	151 (2.32% [1.95, 2.68])	5669 (16.93% [16.53, 17.33])	5622 (16.88% [16.48, 17.28])	111 (3.63% [2.97, 4.30])	64 (2.23% [1.69, 2.77])
Missing	1278 (4.68% [4.43, 4.94])	172 (2.77% [2.36, 3.18])	150 (2.30% [1.94, 2.67])	1450 (4.33% [4.11, 4.55])	1411 (4.24% [4.02,4.45])	105 (3.44% [2.79, 4.08])	66 (2.30% [1.75, 2.85])
**Indigenous Regions**							
501 (Broome)	1876 (6.88% [6.58, 7.18])	146 (2.35% [1.97, 2.73])	141 (2.16% [1.81, 2.52])	2022 (6.04% [5.78, 6.29])	1993 (5.98% [5.73, 6.24])	57 (1.87% [1.39, 2.34])	28 (0.97% [0.62, 1.33])
502 (Geraldton)	2903 (10.64% [10.28, 11.01])	289 (4.65% [4.13, 5.18])	231 (3.55% [3.10, 3.99])	3192 (9.53% [9.22, 9.85])	3089 (9.27% [8.96, 9.59])	188 (6.15% [5.30, 7.00])	85 (2.96% [2.34, 3.58])
503 (Kalgoorlie)	2099 (7.69% [7.38, 8.01])	286 (4.61% [4.08, 5.13])	288 (4.42% [3.92, 4.92])	2385 (7.12% [6.85, 7.40])	2362 (7.09% [6.82, 7.37])	147 (4.81% [4.05, 5.57])	124 (4.32% [3.57, 5.06])
504 (Kununurra)	2337 (8.57% [8.23, 8.90])	50 (0.81% [0.58, 1.03])	47 (0.72% [0.52, 0.93])	2387 (7.13% [6.85, 7.40])	2372 (7.12% [6.85, 7.40])	36 (1.18% [0.80, 1.56])	21 (0.73% [0.42, 1.04])
505 (Perth)	9419 (34.53% [33.96, 35.09])	3806 (61.29% [60.08, 62.50])	4388 (67.34% [66.20, 68.48])	13225 (39.49% [38.97, 40.01])	13585 (40.79% [40.26, 41.32])	1650 (53.99% [52.23, 55.76])	2010 (69.96% [68.29, 71.64])
506 (South Hedland)	2625 (9.62% [9.27, 9.97])	280 (4.51% [3.99, 5.02])	225 (3.45% [3.01, 3.90])	2905 (8.67% [8.37, 8.98])	2828 (8.49% [8.19, 8.79])	160 (5.24% [4.45, 6.03])	83 (2.89% [2.28, 3.50])
507 (South-Western)	3735 (13.69% [13.28, 14.10])	1274 (20.52% [19.51, 21.52])	1126 (17.28% [16.36, 18.20])	5009 (14.96% [14.58, 15.34])	4738 (14.23% [13.85, 14.60])	762 (24.93% [23.40, 26.47])	491 (17.09% [15.71, 18.47])
508 (West-Kimberley)	1867 (6.84% [6.54, 7.14])	40 (0.64% [0.45, 0.84])	30 (0.46% [0.30, 0.62])	1907 (5.69% [5.45, 5.94])	1887 (5.67% [5.42, 5.91])	32 (1.05% [0.69, 1.41])	12 (0.42% [0.18, 0.65])
Missing	418 (1.53% [1.39, 1.68])	39 (0.63% [0.43, 0.82])	40 (0.61% [0.42, 0.80])	457 (1.36% [1.24, 1.49])	452 (1.36% [1.23, 1.48])	24 (0.79% [0.47, 1.10])	19 (0.66% [0.36, 0.96])

Supplementary Table 4: Perinatal outcomes (number, n, and proportion, %) of the children identified as Aboriginal and/or Torres Strait Islander by each identification method (A, B, C – shaded cells), and the additional children identified compared to Methods A, B and C.

	**Method A: Child ISF (n=27,279) n (%, [95%CI])**	**Child/parent/grand- parent ISF** (not by child ISF) **B not A (n=6210) n (%, [95%CI])**	**Perinatal Definition** (not by child ISF) **C not A (n=6516) n (%, [95%CI])**	**Method B: Child or parent or grandparent ISF (n=33,489) n (%, [95%CI])**	**Method C: Perinatal Child MNS or Birth reg (Parent or child* status) (n=33,306) n (%, [95%CI])**	**Child/parent/grand-parent ISF** (not in Perinatal Definition) **B not C (n=3056) n (%, [95%CI])**	**Perinatal Definition** (not in child/parent/grandparent ISF) **C not B (n=2873) n (%, [95%CI])**
**Data available**							
Midwives Notification record	27233 (99.83% [99.78, 99.88])	6206 (99.94% [99.87, 100.00])	6512 (99.94% [99.88, 100.00])	33439 (99.85% [99.81, 99.89])	33256 (99.85% [99.81, 99.89])	3054 (99.93% [99.84, 100.00])	2871 (99.93% [99.83, 100.00])
Birth registration record	24213 (88.76% [88.39, 89.14])	6139 (98.86% [98.59, 99.12])	6470 (99.29% [99.09, 99.50])	30352 (90.63% [90.32, 90.94])	30293 (90.95% [90.65, 91.26])	2921 (95.58% [94.85, 96.31])	2862 (99.62% [99.39, 99.84])
**Gestational Age (GA), weeks**							
Mean (SD)	38.1 (2.9)	38.6 (2.3)	38.6 (2.2)	38.2 (2.8)	38.2 (2.8)	38.5 (2.4)	38.6 (2.1)
Missing	130 (0.5%)	9 (0.1%)	11 (0.2%)	139 (0.4%)	141 (0.4%)	<5 (<0.2%)	6 (0.2%)
**Infant Birthweight (g)**							
Mean (SD)	3116.6 (713.2)	3313.6 (613.4)	3321.8 (602.7)	3153.1 (699.9)	3156.1 (698.2)	3300.0 (631.8)	3343.7 (593.2)
Missing	63 (0.2%)	8 (0.1%)	5 (0.1%)	71 (0.2%)	68 (0.2%)	5 (0.2%)	<5 (<0.2%)
**Preterm birth**							
Extremely Preterm (<28 weeks)	493 (1.81% [1.65, 1.97])	55 (0.89% [0.65, 1.12])	44 (0.68% [0.48, 0.87])	548 (1.64% [1.50, 1.77])	530 (1.59% [1.46, 1.73])	<35 (<1.1% [0.71, 1.45])	15 (0.52% [0.26, 0.79])
Very Preterm (28-32 weeks)	469 (1.72% [1.57, 1.87])	53 (0.85% [0.62, 1.08])	66 (1.01% [0.77, 1.26])	522 (1.56% [1.43, 1.69])	529 (1.59% [1.45, 1.72])	25 (0.82% [0.50, 1.14])	32 (1.11% [0.73, 1.50])
Moderate -Late Preterm 32-37 weeks	3244 (11.89% [11.51, 12.28])	455 (7.33% [6.68, 7.97])	467 (7.17% [6.54, 7.79])	3699 (11.05% [10.71, 11.38])	3644 (10.94% [10.61, 11.28])	251 (8.21% [7.24, 9.19])	196 (6.82% [5.90, 7.74])
Term (>37 weeks)	22943 (84.10% [83.67, 84.54])	5638 (90.79% [90.07, 91.51])	5928 (90.98% [90.28, 91.67])	28581 (85.34% [84.97, 85.72])	28462 (85.46% [85.08, 85.83])	2743 (89.76% [88.68, 90.83])	2624 (91.33% [90.30, 92.36])
Missing	130 (0.48% [0.39, 0.56])	9 (0.14% [0.05, 0.24])	11 (0.17% [0.07, 0.27])	139 (0.42% [0.35, 0.48])	141 (0.42% [0.35, 0.49])	<5 (<0.2% [0.00, 0.26])	6 (0.21% [0.04, 0.38])
**Live born infant characteristics**							

Apgar score of >=9 at 1 min	15394 (57.33% [56.74, 57.92])	3677 (59.69% [58.47, 60.92])	3963 (61.16% [59.97, 62.34])	19071 (57.77% [57.24, 58.30])	19090 (58.11% [57.58, 58.65])	1761 (58.25% [56.50, 60.01])	1780 (62.24% [60.46, 64.01])
Low birth weight (<2500g)	3634 (13.53% [13.12, 13.94])	431 (7.00% [6.36, 7.64])	440 (6.79% [6.18, 7.40])	4065 (12.31% [11.96, 12.67])	4022 (12.24% [11.89, 12.60])	227 (7.51% [6.57, 8.45])	184 (6.43% [5.53, 7.33])
SGA (<10th percentile)	4414 (16.44% [15.99, 16.88])	639 (10.37% [9.61, 11.13])	629 (9.71% [8.99, 10.43])	5053 (15.31% [14.92, 15.69])	4976 (15.15% [14.76, 15.54])	343 (11.35% [10.22, 12.48])	266 (9.30% [8.23, 10.37])

*Child status where available, majority complete from 2007 on Birth registration and 2012 on MN. SD (standard deviation).
